# Head-to-Head Comparison of Meril Myval Series Balloon-Expandable and Abbott Portico Series Self-Expanding Transcatheter Aortic Valves—A Single-Center Experience

**DOI:** 10.3390/medicina61081419

**Published:** 2025-08-06

**Authors:** Matjaž Bunc, Gregor Verček, Luka Vitez, Primož Holc, Klemen Steblovnik, Miha Šušteršič

**Affiliations:** 1 Department of Cardiology, University Medical Centre Ljubljana, Zaloska 7, 1000 Ljubljana, Slovenia; 2 Institute of Pathological Physiology, Faculty of Medicine, University of Ljubljana, Vrazov trg 2, 1000 Ljubljana, Slovenia

**Keywords:** aortic stenosis, TAVI, TAVR, transcatheter heart valve, balloon-expandable valve, self-expanding valve, intra-annular valve

## Abstract

*Background and Objectives*: Transcatheter heart valve (THV) selection is challenging as self-expanding valves (SEVs) are associated with lower post-procedural mean aortic gradients, while balloon-expandable valves (BEVs) have lower rates of paravalvular leak (PVL) and permanent pacemaker implantation (PPI). We aimed to compare the 30-day and 1-year outcomes following Myval BEV (Meril Life Sciences, Vapi, Gujarat, India) and intra-annular Portico SEV (Abbott, St. Paul, MN, USA) implantation. *Materials and Methods*: We retrospectively analyzed the data from the all-comer TAVI registry of the University Medical Centre Ljubljana, Slovenia, from October 2017 to August 2023. Safety and efficacy outcomes following Myval BEV and Portico SEV implantation were compared overall and after propensity score matching. *Results*: Of the total 1152 THVs implanted, 97 patients (8%) received a Myval BEV and 47 (4%) a Portico SEV. After propensity score matching, there were no significant differences between the two patient cohorts regarding 30-day (Myval 0.0% vs. Portico 2.9%, *p* = 1.000) and 1-year mortality (Myval 0.0% vs. Portico 5.9%, *p* = 0.492). Likewise, the rates of new PPI, device failure (mean aortic gradient and more than mild PVL), and periprocedural in-hospital complications were comparable between the two groups. *Conclusions*: In this retrospective analysis of two intra-annular THVs, the Myval BEV was associated with comparable short- and mid-term outcomes as the Portico SEV.

## 1. Introduction

Transcatheter aortic valve implantation (TAVI) has emerged as the preferred treatment for patients with severe aortic stenosis who are at high or prohibitive surgical risk, particularly among older populations [1,2]. Over the past decade, advances in transcatheter heart valve (THV) technologies have significantly expanded the range of options available for TAVI procedures. Among these, two main THV types have become widely used: balloon-expandable valves (BEVs) and self-expanding valves (SEVs). Choosing the optimal THV for a given patient remains complex and is influenced by anatomical, procedural, and device-specific factors. BEVs have been associated with reduced rates of paravalvular leak (PVL) and permanent pacemaker implantation (PPI), although they may produce higher mean trans-prosthetic gradients compared to SEVs [3]. Despite the increasing availability of various THVs, direct head-to-head comparisons between different valve platforms remain limited, particularly in a prospective randomized setting [3,4,5,6,7,8].

The Myval THV (Meril Life Sciences, Vapi, Gujarat, India) is a relatively recent addition to the BEV category. It features a unique hybrid honeycomb nickel–cobalt frame and bovine pericardial leaflets treated to resist calcification [9]. One notable advantage of the Myval system is its wide size matrix—including intermediate and extra-large options—which allows for more precise sizing, reducing the risks of PVL and PPI due to under- or over-sizing [10,11]. Clinical studies have confirmed the safety and effectiveness of the Myval THV in patients with severe native valve aortic stenosis, as well as in challenging settings such as in patients with large annuli, bicuspid valves, and valve-in-valve procedures [11,12,13].

Additionally, the Myval THV has been compared with other contemporary BEVs and SEVs in the LANDMARK randomized trial, demonstrating its non-inferiority for the 30-day composite safety and efficacy outcome [7]. The COMPARE-TAVI trial, the largest randomized study comparing two balloon-expandable THVs in an all-comers population, demonstrated that the Myval THV series is non-inferior to the SAPIEN 3 series (Edwards Lifesciences, Irvine, CA, USA) for the primary composite safety and efficacy endpoint at 1-year follow-up, supporting its generalizability to routine clinical practice [8]. Myval THV was also compared with the supra-annular Evolut series SEVs (Medtronic, Minneapolis, MN, USA) in retrospective single-center observational studies [14,15]. Additionally, in the recent retrospective multi-center MYLAND study, the Myval THV was compared to SAPIEN BEV in the German patient population, demonstrating comparable performance of the two THV systems [16]. In contrast, the Portico SEV (Abbott, St. Paul, MN, USA) is a well-established self-expanding intra-annular valve with a nitinol frame and bovine pericardial leaflets [6]. Moreover, it has established clinical performance, making it a relevant and mechanistically comparable THV. It is designed for flexibility and ease of deployment in various anatomies. Its latest iteration, the Navitor THV, incorporates the NaviSeal cuff to reduce PVL risk [17]. However, in the PORTICO IDE trial, the Portico valve showed higher rates of the primary safety endpoint at 30 days compared with other commercially available valves [6].

In this head-to-head, all-comer, single-center registry study, we chose to compare the Myval and Portico valves, as this represents one of the first direct comparisons between the two platforms. While the Myval THV has been evaluated against Evolut and SAPIEN valves in the LANDMARK and COMPARE-TAVI randomized trials, comparative data with the Portico valve remain lacking [7,8]. Additionally, both devices share an intra-annular design but differ fundamentally in their deployment mechanisms—Myval being balloon-expandable and Portico self-expanding, offering a mechanistically relevant basis for comparison.

## 2. Materials and Methods

This was a retrospective all-comer single-center observational registry study of patients who underwent TAVI with either a Myval BEV (Meril Life Sciences, Vapi, Gujarat, India) or Portico SEV (Abbott, St. Paul, MN, USA) at the University Medical Centre Ljubljana, Slovenia, between October 2017 and August 2023. All patients underwent standard pre-procedural screening according to local protocol and were discussed by the institutional multidisciplinary valvular heart team, which approved the indication for TAVI according to the current guidelines [1]. Ultimate THV selection was at the interventional cardiologist’s discretion. The correct sizes of the THVs were determined by the annular dimensions provided by multi-slice computer tomography. TAVI was performed according to local standards and manufacturers’ instructions. The decision to perform balloon pre- or post-dilatation was made by the interventional cardiologists performing the procedure. The transfemoral route was the default vascular access site, while percutaneous closure was the default closure method. After the procedure, patients were monitored in an intensive care unit for at least 12 to 24 h. Patients underwent routine pre-discharge transthoracic echocardiography for THV hemodynamics and cardiac function assessment, and periodic outpatient clinic follow-up.

Pre-procedural, procedural, and post-procedural data were collected prospectively in a dedicated institutional TAVI registry and analyzed retrospectively. Endpoints were assessed following the Valve Academic Research Consortium (VARC)-3 guidelines [18]. The primary outcome of interest was 30-day and 1-year mortality. Secondary outcomes were the rates of more than mild PVL, new PPI, complications (cardiac, access site, or neurological complications), major or life-threatening bleeding, acute kidney injury after TAVI, and post-procedural mean aortic gradients.

Continuous variables are shown as mean and standard deviation for normally distributed variables or median and interquartile range (IQR) for non-normally distributed variables. Nominal variables are presented as numbers and percentages. Between-group comparison was performed with the two-sample *t*-test or the Mann–Whitney U test in the case of quantitative variables and with the Chi-square/Fisher’s exact test for qualitative variables, as appropriate. The paired t-test and repeated measures two-way ANOVA were used for post hoc analysis to evaluate the significant differences between pre- and post-procedural hemodynamic data within each group. Binary logistic regression analysis was performed to test the association of THV type with the incidence of post-procedural PPI. Survival curves were assessed with the Kaplan–Meier method, and survival distributions of two or more independent groups were compared using the log-rank test. Statistical analysis was performed for the whole patient cohort and after propensity score matching. The distribution of the data was tested with the Shapiro–Wilk test. Propensity matching was performed using nearest neighbor matching with baseline parameters of age, sex, body mass index, body surface area, creatinine, left ventricular ejection fraction, mean aortic gradient, effective orifice area, Society of Thoracic Surgeons (STS) score, New York Heart Association (NYHA) functional class, and conduction disturbances. Baseline characteristics are presented with descriptive statistics. A *p*-value < 0.05 was considered statistically significant. Statistical analysis was performed using R Studio (v4.3.3).

## 3. Results

### 3.1. Included Patients

**Figure 1:**
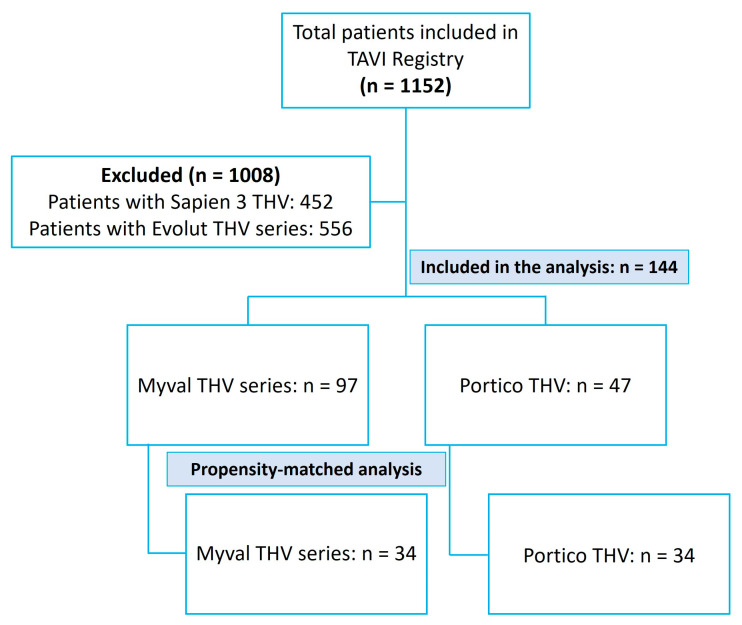
Study outline.

Between October 2017 and August 2023, a total of 1152 THVs were implanted. Out of them, 1008 patients were excluded—452 received the SAPIEN 3 THV and 556 received the Evolut THV. The remaining 144 patients underwent TAVI with either the Myval THV (n = 97) or the Portico THV (n = 47). Three patients received the newer Navitor iteration of the Portico valve. After propensity matching, there were 34 patients in each group (Myval: n = 34, Portico: n = 34), ensuring balanced baseline characteristics for comparative analysis (Figure 1).

### 3.2. Baseline Characteristics

A comparison of baseline characteristics of these unmatched patients showed important differences in several variables, including age, sex, body mass index, body surface area, creatinine, left ventricular ejection fraction, mean aortic gradient, effective orifice area, STS score, NYHA functional class, and conduction disturbances. In the propensity-matched cohort (n = 68), baseline characteristics were generally balanced between the Myval and Portico groups. Median age (Myval: 80.9 vs. Portico: 82.8 years, *p* = 0.081), body mass index (Myval: 26.5 vs. Portico: 27.9 kg/m^2^, *p* = 0.112), body surface area (Myval: 1.8 vs. Portico: 1.85 m^2^, *p* = 1.000), and serum creatinine levels (Myval: 108.5 vs. Portico: 98.3 µmol/L, *p* = 0.295) were comparable. The Myval group had a numerically higher proportion of male patients (Myval: 61.8% vs. Portico: 44.1%, *p* = 0.224). Both groups predominantly presented with severe degenerative aortic stenosis (97.1%) and had similar procedural indications. The EuroScore II was significantly higher in the Myval group (Myval: 4.9 vs. Portico: 3.2%, *p* = 0.044), while the median STS scores were similar. Left ventricular ejection fraction was lower in the Myval group (Myval: 51.7% vs. Portico: 60.1%, *p* = 0.005). Other echocardiographic and anatomical parameters, including valve area, annular dimensions, and systolic pulmonary pressure, showed no significant differences. Baseline conduction abnormalities were comparable, except for a higher incidence of left bundle branch block in the Myval group (Myval: 23.5% vs. Portico: 2.9%, *p* = 0.027). NYHA class distribution was similar across both cohorts (Table 1).

### 3.3. Procedural Characteristics

TAVI was performed via the transfemoral percutaneous route in all but one patient in the Portico THV cohort, in whom surgical cutdown was required. Access site closure was performed percutaneously in most patients, whereas surgical closure was required in three patients (3.1%) following Myval THV and in one patient (2.1%) following Portico THV implantation (Supplementary Table S1). Most TAVI procedures were performed under conscious sedation (Myval THV 99.0% vs. Portico THV 80.0% overall, *p* < 0.0001). Balloon pre-dilatation was performed in 7 patients (7.2%) in the Myval THV cohort and 44 patients (95.6%) in the Portico THV cohort (*p* < 0.0001). Post-dilatation was performed in 3 patients (3.1%) in the Myval THV cohort and 16 patients (34.8%) after Portico THV implantation (*p* < 0.0001). The difference remained significant even after propensity score matching (Supplementary Table S1).

### 3.4. Primary Outcomes

In the unmatched cohort, 30-day mortality was similar between the two groups (Myval: 2.1% vs. Portico: 2.2%, *p* = 1.000), while 1-year all-cause mortality was 5.2% (5/96) in the Myval group and 7.0% (3/43) in the Portico group (*p* = 0.703). Two patients in the Myval group died before hospital discharge (2.2%), whereas no such deaths were reported in the Portico group (*p* = 1.000). No procedural deaths occurred in either group.

**Figure 2:**
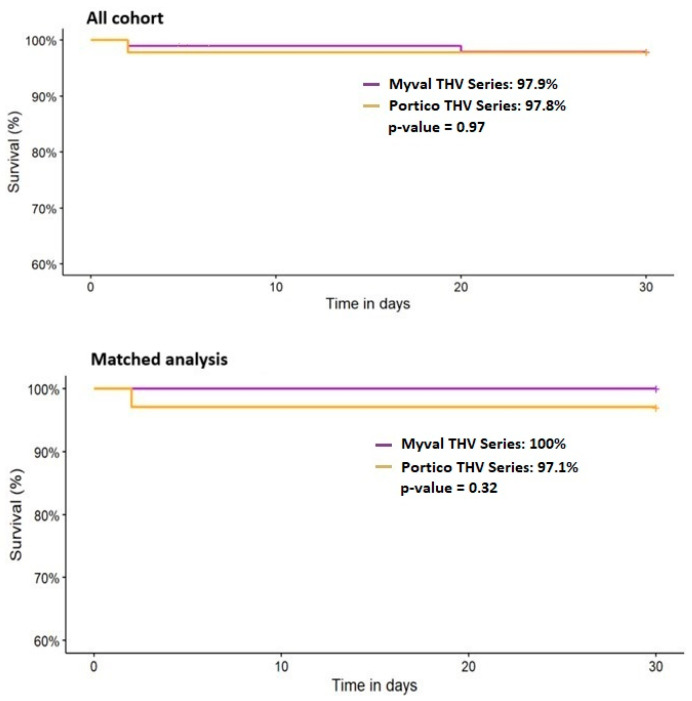
A 30-day mortality for all patients (**top**) and the matched cohorts (**bottom**). THV—transcatheter heart valve.

**Figure 3:**
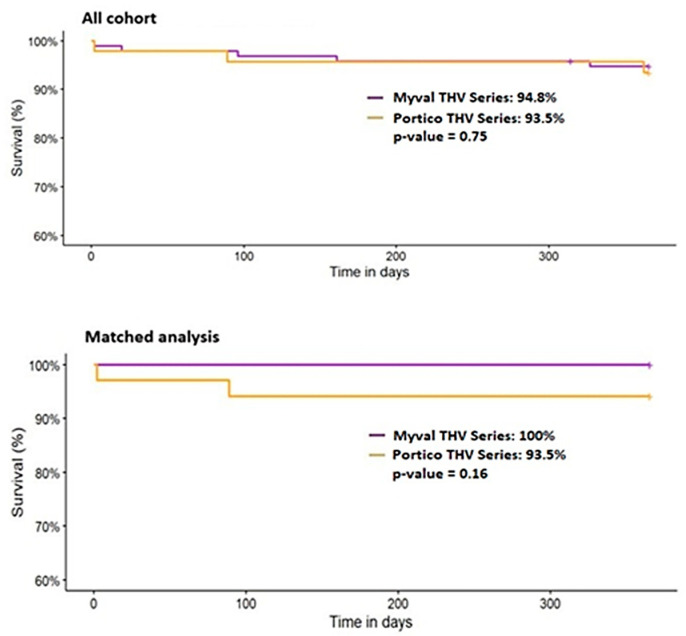
One-year mortality for all patients (**top**) and the matched cohorts (**bottom**). THV—transcatheter heart valve.

After propensity-score matching, 30-day mortality rates were 0.0% vs. 2.9% (*p* = 1.000) for the Myval and Portico groups, and 1-year mortality was 0.0% (0/33) in the Myval group and 5.9% (2/34) in the Portico group (*p* = 0.492), respectively. There were no deaths before hospital discharge or procedural deaths in either matched group. Although mortality was numerically lower with the Myval THV at both 30 days and 1 year, the differences were not statistically significant (Table 2, Figure 2 and Figure 3).

### 3.5. Secondary Outcomes

In the matched cohorts, moderate paravalvular leak (PVL) occurred in two patients (6.3%) in the Portico group, whereas no moderate or severe PVL was observed in the Myval group. Mild PVL was seen in 28.1% and 26.5% of patients in the Portico and Myval groups, respectively. The proportion of patients with none or trace PVL was numerically higher in the Myval group (Myval: 73.5% vs. Portico: 65.6%), although these differences did not reach statistical significance (*p* = 0.373) (Table 2). Permanent pacemaker implantation (PPI) was required in five patients (15.6%) in the Portico group and three patients (9.1%) in the Myval group (*p* = 0.475) (Table 2). However, additional multivariate logistic regression analysis confirmed that valve type was not significantly associated with the risk of PPI (odds ratio: 0.329, *p*-value: 0.247, Supplementary Table S2).

Neurological complications were rare and occurred only in the Myval group (one patient, 3.0%), while none were reported in the Portico cohort (Table 2; Supplementary Table S3). Cardiac complications were observed in three patients (9.1%) in the Portico group and in one patient (3.0%) in the Myval group (*p* = 0.613). Specifically, the Portico group experienced one case of tamponade, two cases of improper valve position, and no valve embolization or annular rupture. In contrast, the Myval group had one case of peri-procedural myocardial infarction (MI) within 72 h post-TAVI, but no structural complications.

Bleeding complications were low and comparable between groups. Major bleeding occurred in two patients (5.9%) in the Portico group and in one patient (2.9%) in the Myval group (*p* = 1.000). No life-threatening bleeding was reported in either group (Table 2). Post-procedural renal function, as measured by serum creatinine levels, was similar between groups (median 87.5 µmol/L [IQR: 75.5–110.8] for Myval vs. 90.0 µmol/L [IQR: 76.8–111.5] for Portico; *p* = 0.908), indicating no significant difference in acute kidney injury post-TAVI (Table 2).

Overall, no significant differences were observed in secondary clinical outcomes between the Myval and Portico groups.

### 3.6. Hemodynamic Outcomes

The post-procedural mean trans-prosthetic gradients were not significantly different in the Myval and Portico groups (Myval: 8.9 ± 2.5 mmHg vs. Portico: 8.1 ± 4.7 mmHg; *p* = 0.398) (Table 3). Similarly, the aortic valve area (AVA) was similar among the groups (Myval: 1.9 ± 0.4 cm^2^ vs. Portico: 1.8 ± 0.5 cm^2^, *p* = 0.372), and peak aortic velocity (Vmax) comparable in both groups (Myval: 1.9 ± 0.3 m/s vs. Portico: 1.9 ± 0.4 m/s, *p* = 1.000). Notably, left ventricular ejection fraction (LVEF) post-TAVI was significantly higher in the Portico group (61.1 ± 11.4%) compared to the Myval group (54.1 ± 14.1%; *p* = 0.033). This probably reflects pre-existing baseline differences.

Pre- and post-procedural hemodynamic parameters are presented in Supplementary Table S4. Repeated measures two-way ANOVA analysis confirmed that there were no significant differences between the groups across serial hemodynamic measurements (Supplementary Table S5). Overall, both THVs demonstrated favorable post-procedural hemodynamic profiles with low mean gradients and adequate valve areas.

## 4. Discussion

The main finding of this retrospective, all-comer, single-center registry study is that the intra-annular balloon-expandable Myval transcatheter heart valve (THV) demonstrated comparable 30-day and 1-year outcomes to the intra-annular self-expanding Portico THV. Notably, there were no significant differences between the two cohorts in the need for new permanent pacemaker implantation (PPI), incidence of more than mild post-procedural paravalvular leak (PVL), or severe PVL, which did not occur in either group. Additionally, the rates of other post-procedural complications—including major bleeding and changes in serum creatinine—were similarly low between groups.

Propensity-score matching yielded two comparable patient cohorts. However, there were still some differences, such as a much lower starting left ventricular ejection fraction (LVEF) and a higher EuroScore II in the Myval group. Despite these differences, the short- and mid-term clinical outcomes remained equivalent between the groups, underscoring the robustness of both devices in varied patient risk profiles.

The substantially higher rates of balloon pre-dilatation (96%) and post-dilatation (35%) in the Portico group, compared to the Myval group (7% and 3%, respectively), highlight key procedural differences driven by device design. The Portico SEV, with its lower initial radial force and self-expanding nature, often necessitates adjunctive ballooning to ensure optimal expansion and positioning [19,20]. In contrast, the Myval THV is balloon-deployed, offers a higher radial force upon implantation, and has a sealing cuff at the bottom of the frame, thereby improving sealing and reducing the risk for PVL [21]. This is reflected in lower rates of balloon-post-dilatation as compared with other contemporary THVs, particularly SEVs [7]. However, these procedural differences did not result in adverse patient outcomes, as no significant differences in complication rates were observed in our study.

The pre-matching gender imbalance (53.6% male in the Myval group vs. 66.0% female in the Portico group) was mitigated post-matching; however, the potential impact of sex-specific anatomical differences warrants acknowledgment. Women undergoing TAVI are known to have smaller aortic annuli, left ventricular outflow tracts, and iliofemoral arteries compared to men, which may affect valve sizing, access strategy, and procedural outcomes [22]. However, a sex-stratified sub-analysis did not show notable differences for the primary and secondary outcomes between genders following TAVI in both matched groups, except for a higher rate of new LBBB in females in the Myval group (Supplementary Table S6).

Cardiac complications occurred in 3% of the Myval group and 9.1% of the Portico group, although this difference was not statistically significant (*p* = 0.613). The nature of complications differed: the Myval group experienced one peri-procedural myocardial infarction, while the Portico group experienced three cardiac complications, of which two of them are THV positioning-related events and one case of cardiac tamponade. These differences may be attributed to valve design—SEVs are more prone to embolization and migration [23], whereas BEVs may more frequently contribute to acute coronary artery obstruction [24]. However, in our cohort, myocardial infarctions in the Myval group were related to pre-existing coronary artery disease or embolic events rather than THV-mediated coronary obstruction. For reference, the LANDMARK trial also reported only one case of coronary obstruction requiring intervention among 379 Myval recipients [7].

Both valves yielded significant improvements in hemodynamic parameters post-TAVI. After matching, the mean trans-prosthetic gradients were low and comparable between groups (Myval: 8.9 ± 2.5 mmHg vs. Portico: 8.1 ± 4.7 mmHg; *p* = 0.398), as were the valve areas and peak aortic velocities. Although Portico recipients had significantly higher post-procedural LVEF (Portico: 61.1% vs. Myval: 54.1%, *p* = 0.033), this finding probably reflects pre-existing baseline differences (Supplementary Table S4).

To date, few direct comparisons between intra-annular THVs exist. Most existing literature either evaluates BEVs and SEVs as broad categories or compares supra-annular devices. The LANDMARK randomized trial demonstrated non-inferiority of Myval versus SAPIEN and Evolut THVs at 30 days, with 2% mortality, 3% moderate PVL, <1% severe PVL, and 15% new PPI [7]—results consistent with our findings.

In the EVAL registry, Myval implantation was associated with a significantly lower incidence of more than mild PVL at 30 days compared to Evolut R (Myval: 3.45% vs. Evolut R: 14.8%, *p* = 0.0338), although differences in PPI (Myval: 11% vs. Evolut R: 24.2%) and mean gradients were not statistically significant [14]. These advantages persisted at 6 months, with lower PVL (Myval: 6.9% vs. Evolut R: 19.8%, *p* = 0.0396) and PPI rates (Myval: 11% vs. Evolut R: 27.5%, *p* = 0.02). A similar propensity-matched study by Halim et al. confirmed lower 30-day PPI rates with Myval compared to Evolut series THVs (Myval: 4% vs. Evolut: 15%, *p* = 0.01), with similar mean gradients and PVL [15].

The MYLAND study comparing Myval and SAPIEN valves showed comparable clinical outcomes, though Myval was associated with a higher rate of major vascular complications (Myval: 6.7% vs. SAPIEN: 1.9%, *p* = 0.02), which was attributed to limited experience and larger vascular access of the Myval platform [16]. A recent review further supports Myval’s favorable safety profile and efficacy across anatomically challenging settings such as large annuli, bicuspid valves, and valve-in-valve procedures [11].

Next, we observed numerically higher rates of moderate PVL and PPI in the Portico group, though not statistically significant. These trends may reflect the lower radial force and prolonged expansion characteristics of the self-expanding Portico frame, which can impact annular sealing and conduction tissue compression. Notably, the PORTICO-IDE trial reported a 30-day PPI rate of 28.1% and more than mild PVL in 6.3% of patients in the as-treated population, followed by 7.5% at 1 year and 5.2% at 2 years [6]. In contrast, our study showed lower rates for both outcomes, which may reflect improvements in deployment technique and a lower-risk patient cohort [25].

Several limitations merit consideration. First, the retrospective and non-randomized nature of the study introduces potential for selection and reporting bias, despite the use of propensity-score matching. Second, the relatively small sample size and inclusion of patients with varying indications—such as isolated aortic regurgitation or valve-in-valve cases—may limit generalizability. Accordingly, due to the small matched sample size and low event rates, the study remains underpowered to detect significant differences in rare complications such as device embolization or myocardial infarction, even under optimistic assumptions. Third, the absence of a core echocardiographic laboratory may have introduced inter-observer variability in hemodynamic measurements and particularly in the assessment of PVL. However, all echocardiographic assessments were conducted using standardized institutional protocols by experienced sonographers under the supervision of the same structural heart team, ensuring consistency in imaging acquisition and interpretation. Lastly, institutional experience and operator familiarity with specific THVs may have influenced procedural decisions and outcomes.

Nonetheless, this study contributes important real-world evidence by providing one of the first direct comparisons between two intra-annular THVs, supporting their clinical equivalence in selected TAVI patients.

## 5. Conclusions

In this retrospective all-comer single-center registry study, we compared the 30-day and 1-year efficacy and safety of the Myval BEV with the established intra-annular Portico SEV, demonstrating comparable short- and mid-term mortality outcomes. There were also no significant differences in secondary outcomes (PVL, PPI, complications, bleeding, acute kidney injury after the procedure) between the unmatched and matched cohorts. However, the study remains underpowered to detect significant differences in rare complications. Therefore, further multi-center, prospective randomized trials with larger patient populations and newer valve iterations are warranted to validate these results and support device selection strategies in diverse anatomical and clinical contexts.

## Figures and Tables

**Table 1:** Baseline patient characteristics overall and after propensity-score matching.

Baseline Characteristics	Overall Cohort			Matched Cohort		
	Myval (n = 97)	Portico (n = 47)	*p*-Value	Myval (n = 34)	Portico (n = 34)	*p*-Value
Age (Years), Median (IQR)	81.0 (77.5, 86.2) (n = 97)	82.9 (79.8, 85.3) (n = 47)	0.248	80.9 (75.6, 84.9) (n = 34)	82.8 (79.7, 85.2) (n = 34)	0.081
Sex, n (%)	n = 97	n = 47		n = 34	n = 34	
Male	52 (53.6)	16 (34.0)	0.043	21 (61.8)	15 (44.1)	0.224
Female	45 (46.4)	31 (66.0)		13 (38.2)	19 (55.9)	
BMI (kg/m^2^), Median (IQR)	28.0 (24.8, 31.8) (n = 97)	27.7 (25.7, 31.9) (n = 45)	0.393	26.5 (24.8, 29.3) (n = 34)	27.9 (25.7, 31.7) (n = 34)	0.112
Body surface area (m^2^), Median (IQR)	1.8 (1.7, 2.1) (n = 97)	1.8 (1.7, 2.0) (n = 45)	0.322	1.8 (1.7, 2.0) (n = 34)	1.85 (1.7, 1.98) (n = 34)	1.000
Creatinine (µmol/L), Mean ± SD	102.3 ± 68.6 (n = 97)	96.5 ± 26.5 (n = 45)	0.581	108.5 ± 50.9 (n = 34)	98.3 ± 23.6 (n = 34)	0.295
Indication, n (%)	n = 97	n = 47		n = 34	n = 34	
Stenosis	96 (99.0)	46 (97.9)	0.548	33 (97.1)	33 (97.1)	1.000
Regurgitation	1 (1.0)	1 (2.1)		1 (2.9)	1 (2.9)	
Etiology, n (%)	n = 97	n = 47		n = 34	n = 34	
Degenerative	91 (93.8)	44 (93.6)	1.000	32 (94.1)	32 (94.1)	1.000
Rheumatic	0 (0)	0 (0)		0 (0)	0 (0)	
Valve-in-Valve (ViV)	6 (6.2)	3 (6.4)		2 (5.9)	2 (5.9)	
EuroScore II, Median (IQR)	3.8 (2.3, 6.3) (n = 96)	3.3 (2.3, 5.0) (n = 41)	0.597	4.9 (3.1, 8.5) (n = 33)	3.2 (2.2, 4.6) (n = 34)	0.044
STS score, Median (IQR)	2.9 (2.1, 4.0) (n = 97)	3.4 (2.9, 4.8) (n = 41)	0.095	4.0 (2.7, 5.4) (n = 34)	3.6 (2.6, 5.1) (n = 34)	0.716
LVEF, (%), Mean ± SD	58.2 ± 13.0 (n = 96)	59.3 ± 10.7 (n = 39)	0.623	51.7 ± 13.1 (n = 34)	60.1 ± 10.0 (n = 34)	0.005
Aortic mean gradient (mmHg), Mean ± SD	45.1 ± 12.5 (n = 96)	43.8 ± 10.7 (n = 41)	0.542	43.7 ± 13.1 (n = 34)	44.0 ± 11.4 (n = 34)	0.929
Aortic valve area (cm^2^), Mean ± SD	0.73 ± 0.19 (n = 95)	0.72 ± 0.19 (n = 42)	0.720	0.74 ± 0.25 (n = 32)	0.74 ± 0.21 (n = 34)	0.280
AV block I	8 (8.3)	6 (12.8)	0.386	3 (8.8)	4 (11.8)	1.000
AV block II	0 (0.0)	0 (0.0)	-	0 (0.0)	0 (0.0)	-
RBBB	8 (8.3)	3 (6.4)	1.000	2 (5.9)	3 (8.8)	1.000
LBBB	10 (10.3)	3 (6.4)	0.547	8 (23.5)	1 (2.9)	0.027
Electrosystolic rhythm	3 (3.1)	5 (10.6)	0.114	3 (8.8)	4 (11.8)	1.000
Atrial fibrillation—slow ventricular response	22 (22.7)	12 (25.5)	0.866	8 (23.5)	10 (29.4)	0.783
Systolic pulmonary artery pressure (mm Hg), Median (IQR)	38.0 (30.8, 44.3) (n = 60)	38.5 (33.3, 48.0) (n = 30)	0.566	42.0 (34.3, 51.3) (n = 18)	38.0 (33.0, 48.0) (n = 29)	0.615
Annular perimeter (mm), Median (IQR)	78.0 (74.2, 83.4) (n = 91)	76.2 (73.6, 81.8) (n = 45)	0.122	78.4 (75.5, 85.3) (n = 31)	76.50 (73.6, 82.4) (n = 33)	0.094
Annular area (mm^2^), Median (IQR)	451.5 (413.8, 524.5) (n = 92)	439.5 (405.3, 487.3) (n = 46)	0.070	465.0 (416.0, 534.0) (n = 31)	439.5 (409.8, 504.5) (n = 34)	0.095
NYHA class before, n (%)	n = 97	n = 46		n = 34	n = 34	
1	4 (4.1)	0 (0.0)	0.275	1 (2.9)	0 (0.0)	0.491
2	17 (17.5)	12 (26.1)		5 (14.7)	9 (26.5)	
3	68 (70.1)	28 (60.9)		23 (67.7)	22 (64.7)	
4	8 (8.3)	6 (13.0)		5 (14.7)	3 (8.8)	

Data is presented as median and interquartile range (IQR) or number and %. BMI—body mass index, IQR—interquartile range, SD—standard deviation, STS—Society of Thoracic Surgeons, LVEF—left ventricular ejection fraction, AV block I—first-degree atrioventricular heart block, AV block II—second-degree atrioventricular heart block, LBBB—left bundle branch block, RBBB—right bundle branch block, NYHA—New York Heart Association.

**Table 2:** Outcomes following Myval and Portico THV implantation, overall and after propensity-score matching.

Outcomes, n (%)	Unmatched Cohort			Matched Cohort		
	Myval (n = 97)	Portico (n = 47)	*p*-Value	Myval (n = 34)	Portico (n = 34)	*p*-Value
Primary outcomes						
1-year mortality	5 (5.2) (n = 96)	3 (7.0) (n = 43)	0.703	0 (0.0) (n = 33)	2 (5.9) (n = 34)	0.492
30-day mortality	2 (2.1) (n = 96)	1 (2.2) (n = 46)	1.000	0 (0.0) (n = 33)	1 (2.9) (n = 34)	1.000
Post-procedural death until hospital discharge	2 (2.2) (n = 93)	0 (0.0) (n = 41)	1.000	0 (0.0) (n = 33)	0 (0.0) (n = 30)	-
Procedural death	0 (0.0)	0 (0.0)	-	0 (0.0)	0 (0.0)	-
Secondary outcomes						
Paravalvular regurgitation	n = 93	n = 45	-	n = 33	n = 32	-
None/Trace	70 (75.3)	31 (68.9)	0.175	25 (73.5)	21 (65.6)	0.373
Mild	22 (23.7)	11 (24.4)		9 (26.5)	9 (28.1)	
Moderate	1 (1.1)	3 (6.7)		0 (0.0)	2 (6.3)	
Severe	0 (0.0)	0 (0.0)		0 (0.0)	0 (0.0)	
Pacemaker implantation after TAVI	10 (10.5) (n = 95)	5 (11.6) (n = 43)	1.000	3 (9.1) (n = 33)	5 (15.6) (n = 32)	0.475
Complications						
Neurological complications	3 (3.2) (n = 94)	0 (0.0) (n = 44)	0.551	1 (3.0) (n = 33)	0 (0.0) (n = 33)	-
Cardiac complications	4 (4.2) (n = 95)	4 (9.3) (n = 43)	0.256	1 (3.0) (n = 33)	3 (9.1) (n = 33)	0.613
New pericardial effusion	0 (0.0)	0 (0.0)	-	0 (0.0)	0 (0.0)	-
Tamponade	0 (0.0)	1 (2.1)	0.326	0 (0.0)	1 (2.9)	1.000
Annular rupture	1 (1.0)	0 (0.0)	1.000	0 (0.0)	0 (0.0)	-
Valve embolization	0 (0)	1 (2.1)	0.326	0 (0.0)	0 (0.0)	-
Improper valve position	0 (0)	2 (4.3)	0.105	0 (0.0)	2 (5.9)	0.493
Conversion to heart surgery	0 (0.0)	1 (2.1)	0.326	0 (0.0)	0 (0.0)	-
Peri-procedural MI (<72 h)	3 (3.1)	0 (0.0)	0.551	1 (2.9)	0 (0.0)	1.000
Spontaneous MI (>72 h)	0 (0.0)	0 (0.0)	-	0 (0.0)	0 (0.0)	-
Bleeding	7 (7.4)	3 (7.0)	1.000	3 (9.1)	3 (9.4)	1.000
Minor bleeding	5 (5.2)	1 (2.1)	0.664	2 (5.9)	1 (2.9)	1.000
Major bleeding	1 (1.0)	2 (4.3)	0.248	1 (2.9)	2 (5.9)	1.000
Life-threatening bleeding	1 (1.0)	0 (0.0)	1.000	0 (0.0)	0 (0.0)	-
Creatinine after (µmol/L), Median (IQR)	60.5 (54.0, 68.0) (n = 92)	62.0 (54.7, 71.0) (n = 43)	0.806	87.5 (75.5, 110.8) (n = 34)	90.0 (76.8, 111.5) (n = 32)	0.908

MI—myocardial infarction; TAVI—transcatheter aortic valve implantation; THV—transcatheter heart valve; IQR—interquartile range.

**Table 3:** Comparison of post-procedural THV hemodynamics.

	Post-Procedure					
	Unmatched Cohort			Matched Cohort		
	Myval (n = 97)	Portico (n = 47)	*p*-Value	Myval (n = 34)	Portico (n = 34)	*p*-Value
Aortic Vmax (m/s), Mean ± SD	2.1 ± 0.5 (n = 93)	2.0 ± 0.4 (n = 41)	0.062	1.9 ± 0.3 (n = 33)	1.9 ± 0.4 (n = 32)	1.000
Aortic mean gradient (mm Hg), Mean ± SD	11.1 ± 5.2 (n = 93)	8.5 ± 4.5 (n = 38)	0.007	8.9 ± 2.5 (n = 33)	8.1 ± 4.7 (n = 29)	0.398
AVA (cm^2^), Mean ± SD	1.8 ± 0.4 (n = 94)	1.8 ± 0.5 (n = 44)	0.517	1.9 ± 0.4 (n = 34)	1.8 ± 0.5 (n = 32)	0.372
LVEF (%), Mean ± SD	59.9 ± 12.5 (n = 86)	61.7 ± 11.2 (n = 40)	0.412	54.1 ± 14.1 (n = 33)	61.1 ± 11.4 (n = 31)	0.033

Data is presented as mean ± SD. AVA—aortic valve area, LVEF—left ventricular ejection fraction, SD—standard deviation, THV—transcatheter heart valve, Vmax—maximal speed of blood in ascending aorta measured with continuous Doppler ultrasound.

## Data Availability

Data available on request from the corresponding author.

## Supplementary Materials

The following supporting information can be downloaded at: https://www.mdpi.com/article/10.3390/medicina61081419/s1, Table S1. Procedural characteristics; Table S2. Binary logistic regression analysis of predictors for permanent pacemaker implantation; Table S3. Post-procedural secondary outcomes; Table S4. Comparison of pre- and post-procedural hemodynamic parameters; Table S5. Analysis of hemodynamic parameters by using repeated measure two-way ANOVA; Table S6. Sex stratified analysis of outcomes post-TAVI.

## References

[1] Vahanian A., Beyersdorf F., Praz F., Milojevic M., Baldus S., Bauersachs J., Capodanno D., Conradi L., De Bonis M., De Paulis R. (2022). 2021 ESC/EACTS Guidelines for the management of valvular heart disease. Eur. Heart J..

[2] Otto C.M., Nishimura R.A., Bonow R.O., Carabello B.A., Erwin J.P., Gentile F., Jneid H., Krieger E.V., Mack M., McLeod C. (2021). 2020 ACC/AHA Guideline for the Management of Patients With Valvular Heart Disease: A Report of the American College of Cardiology/American Heart Association Joint Committee on Clinical Practice Guidelines. Circulation.

[3] Abdel-Wahab M., Mehilli J., Frerker C., Neumann F.J., Kurz T., Tölg R., Zachow D., Guerra E., Massberg S., Schäfer U. (2014). Comparison of balloon-expandable vs. self-expandable valves in patients undergoing transcatheter aortic valve replacement: The CHOICE randomized clinical trial. JAMA.

[4] Tamburino C., Bleiziffer S., Thiele H., Scholtz S., Hildick-Smith D., Cunnington M., Wolf A., Barbanti M., Tchetchè D., Garot P. (2020). Comparison of Self-Expanding Bioprostheses for Transcatheter Aortic Valve Replacement in Patients With Symptomatic Severe Aortic Stenosis: SCOPE 2 Randomized Clinical Trial. Circulation.

[5] Thiele H., Kurz T., Feistritzer H.J., Stachel G., Hartung P., Eitel I., Marquetand C., Nef H., Doerr O., Lauten A. (2020). Comparison of newer generation self-expandable vs. balloon-expandable valves in transcatheter aortic valve implantation: The randomized SOLVE-TAVI trial. Eur. Heart J..

[6] Makkar R.R., Cheng W., Waksman R., Satler L.F., Chakravarty T., Groh M., Abernethy W., Russo M.J., Heimansohn D., Hermiller J. (2020). Self-expanding intra-annular versus commercially available transcatheter heart valves in high and extreme risk patients with severe aortic stenosis (PORTICO IDE): A randomised, controlled, non-inferiority trial. Lancet.

[7] Baumbach A., van Royen N., Amat-Santos I.J., Hudec M., Bunc M., Ijsselmuiden A., Laanmets P., Unic D., Merkely B., Hermanides R.S. (2024). LANDMARK comparison of early outcomes of newer-generation Myval transcatheter heart valve series with contemporary valves (Sapien and Evolut) in real-world individuals with severe symptomatic native aortic stenosis: A randomised non-inferiority trial. Lancet.

[8] Terkelsen C.J., Freeman P., Dahl J.S., Thim T., Nørgaard B.L., Mogensen N.S.B., Tang M., Eftekhari A., Povlsen J.A., Poulsen S.H. (2025). SAPIEN 3 versus Myval transcatheter heart valves for transcatheter aortic valve implantation (COMPARE-TAVI 1): A multicentre, randomised, non-inferiority trial. Lancet.

[9] Testa L., Criscione E., Popolo Rubbio A., Squillace M., Ielasi A., Tespili M., Brambilla N., Bedogni F. (2023). Safety and performance parameters of the Myval transcatheter aortic valve bioprosthesis: The SAPPHIRE prospective registry. Cardiovasc. Revasc. Med..

[10] Seth A., Kumar V., Singh V.P., Kumar D., Varma P., Rastogi V. (2023). Myval: A Novel Transcatheter Heart Valve for the Treatment of Severe Aortic Stenosis. Interv. Cardiol..

[11] Montonati C., Pellegrini D., d’Atri D.O., Pellicano M., Briguglia D., Giannini F., De Blasio G., Guagliumi G., Tespili M., Ielasi A. (2024). A novel balloon-expandable transcatheter aortic valve bioprosthesis: Myval and Myval Octacor. Expert Rev. Cardiovasc. Ther..

[12] Sharma S.K., Rao R.S., Chandra P., Goel P.K., Bharadwaj P., Joseph G., Jose J., Mahajan A.U., Mehrotra S., Sengottovelu G. (2020). First-in-human evaluation of a novel balloon-expandable transcatheter heart valve in patients with severe symptomatic native aortic stenosis: The MyVal-1 study. EuroIntervention.

[13] Kilic T., Ielasi A., Ninios V., Korkmaz L., Panagiotakos D., Yerlikaya G., Ozderya A., Montonati C., Tespili M., Coskun S. (2024). Clinical outcomes of the Myval transcatheter heart valve system in patients with severe aortic valve stenosis: A two-year follow-up observational study. Arch. Med. Sci..

[14] Barki M., Ielasi A., Buono A., Maliandi G., Pellicano M., Bande M., Casilli F., Messina F., Uccello G., Briguglia D. (2022). Clinical Comparison of a Novel Balloon-Expandable Versus a Self-Expanding Transcatheter Heart Valve for the Treatment of Patients with Severe Aortic Valve Stenosis: The EVAL Registry. J. Clin. Med..

[15] Halim J., Rooijakkers M., den Heijer P., El Haddad M., van den Branden B., Vos J., Schölzel B., Meuwissen M., van Gameren M., El Messaoudi S. (2023). Assessing the Novel Myval Balloon-Expandable Valve with the Evolut Valve: A Propensity-Matched Study. J. Clin. Med..

[16] Ubben T., Tigges E., Kim W.K., Holzamer A., Breitenbach I., Sodian R., Rothe J., Hochholzer W., Hakmi S., Neumann F.J. (2024). German Experience with a Novel Balloon-Expandable Heart Valve Prosthesis for Transcatheter Aortic Valve Implantation-Outcomes of the MYLAND (MYvaL germAN stuDy) Study. J. Clin. Med..

[17] Reardon M.J., Chehab B., Smith D., Walton A.S., Worthley S.G., Manoharan G., Sultan I., Yong G., Harrington K., Mahoney P. (2023). 30-Day Clinical Outcomes of a Self-Expanding Transcatheter Aortic Valve: The International PORTICO NG Study. JACC Cardiovasc. Interv..

[18] Généreux P., Piazza N., Alu M.C., Nazif T., Hahn R.T., Pibarot P., Bax J.J., Leipsic J.A., Blanke P., Blackstone E.H. (2021). Valve Academic Research Consortium 3: Updated endpoint definitions for aortic valve clinical research. Eur. Heart J..

[19] Gorla R., De Marco F., Morganti S., Finotello A., Brambilla N., Testa L., Agnifili M.L., Tusa M., Auricchio F., Bedogni F. (2020). Transcatheter aortic valve implantation with the Portico and Evolut R bioprostheses in patients with elliptic aortic annulus. EuroIntervention.

[20] Hameau R., Ancona M.B., Romano V., Ferri L., Bellini B., Russo F., Vella C., Papageorgiu C., Napoli F., Licciardi M. (2025). Management of TAVI Underexpansion with Self-Expanding Valves: A Practical Approach. J. Cardiovasc. Dev. Dis..

[21] Kilic T., Coskun S., Mirzamidinov D., Yilmaz I., Yavuz S., Sahin T. (2024). Myval Transcatheter Heart Valve: The Future of Transcatheter Valve Replacement and Significance in Current Timeline. J. Clin. Med..

[22] Matetic A., Kristić I., Crnčević N., Zanchi J., Domjanović Škopinić T., Baković Kramarić D., Runjić F. (2025). Sex-specific anatomic differences in patients undergoing transcatheter aortic valve implantation: Insights from the ST-TAVI registry. Hell. J. Cardiol..

[23] Kim W.K., Schäfer U., Tchetche D., Nef H., Arnold M., Avanzas P., Rudolph T., Scholtz S., Barbanti M., Kempfert J. (2019). Incidence and outcome of peri-procedural transcatheter heart valve embolization and migration: The TRAVEL registry (TranscatheteR HeArt Valve EmboLization and Migration). Eur. Heart J..

[24] Ojeda S., González-Manzanares R., Jiménez-Quevedo P., Piñón P., Asmarats L., Amat-Santos I., Fernández-Nofrerias E., Valle R.D., Muñoz-García E., Ferrer-Gracia M.C. (2023). Coronary Obstruction After Transcatheter Aortic Valve Replacement: Insights From the Spanish TAVI Registry. JACC Cardiovasc. Interv..

[25] Fontana G.P., Bedogni F., Groh M., Smith D., Chehab B.M., Garrett H.E., Yong G., Worthley S., Manoharan G., Walton A. (2020). Safety Profile of an Intra-Annular Self-Expanding Transcatheter Aortic Valve and Next-Generation Low-Profile Delivery System. JACC Cardiovasc. Interv..

