# Supplementary material for: Head-to-Head Comparison of Meril Myval Series Balloon-Expandable and Abbott Portico Series Self-Expanding Transcatheter Aortic Valves—A Single-Center Experience
Source: Medicina (Kaunas). 2025 Aug 6;61(8):1419. doi: 10.3390/medicina61081419 (PMC12388230; doi:10.3390/medicina61081419)
Supplement: Supplementary file 1 [file medicina-61-01419-s001.zip › medicina-3725155-supplementary.pdf]

Supplementary material

Table S1. Procedural characteristics.

Procedural details<br>n (%)	Overall cohort		p-value	Matched cohort		p-value
	Myval<br>(n = 97)	Portico<br>(n = 47)		Myval<br>(n = 34)	Portico<br>(n = 34)	
Access site	n = 97 (100.0)	n = 47 (100.0)		n = 34 (100.0)	n = 34 (100.0)	
Transfemoral – right	83 (85.6)	33 (70.2)	0.038	28 (82.4)	24 (70.6)	0.392
Transfemoral - right with surgical cutdown	0 (0.0)	0 (0.0)		0 (0.0)	0 (0.0)	
Transfemoral - left	14 (14.4)	13 (27.7)		6 (17.7)	9 (26.5)	
Transfemoral - left with surgical cutdown	0 (0.0)	1 (2.1)		0 (0.0)	1 (2.9)	
Closure device						
1 x Proglide,	8 (8.3)	0 (0.0)	0.010	3 (8.8)	0 (0.0)	0.078
2 x Proglide + AngioSeal	17 (17.5)	5 (10.6)		4 (11.8)	3 (8.8)	
2 x Proglide	8 (8.3)	0 (0.0)		3 (8.8)	0 (0.0)	
1x Proglide + AngioSeal	58 (59.8)	38 (80.9)		22 (64.7)	28 (82.4)	
Conversion from percutaneous to surgical closure	3 (3.1)	1 (2.1)	1.000	1 (2.9)	1 (2.9)	1.000
Sizes of Myval THV implanted	n = 97 (100.0)	-		n = 34 (100.0)	-	
20 mm	0 (0.0)	-	NA	0 (0.0)	-	NA
21.5 mm	0 (0.0)	-	NA	0 (0.0)	-	NA
23 mm	14 (14.4)	-	NA	4 (11.7)	-	NA

24.5 mm	38 (39.2)	-	NA	13 (38.2)	-	NA
26 mm	19 (19.6)	-	NA	5 (14.7)	-	NA
27.5 mm	13 (13.4)	-	NA	7 (20.6)	-	NA
29 mm	7 (7.2)	-	NA	3 (8.8)	-	NA
30.5 mm	1 (1.0)	-	NA	0 (0.0)	-	NA
32 mm	5 (5.2)	-	NA	2 (5.9)	-	NA
Sizes of Portico implanted	-	n = 47 (100.0)		-	n = 34 (100.0)	
23 mm	-	3 (6.4)	NA	-	2 (5.9)	NA
25 mm	-	5 (10.6)	NA	-	3 (8.8)	NA
27 mm	-	22 (46.8)	NA	-	15 (44.1)	NA
29 mm	-	17 (36.2)	NA	-	14 (41.2)	NA
Two TAVI prostheses implanted	2 (2.1)	4 (8.7)	0.089	0 (0.0)	3 (9.1)	0.239
Balloon pre-dilatation	7 (7.2)	44 (95.6)	< 0.001	2 (5.9)	31 (93.9)	< 0.001
Balloon post-dilatation	3 (3.1)	16 (34.8)	< 0.001	1 (2.9)	14 (42.4)	< 0.001
Sedation	n = 97 (100.0)	n = 45 (95.7)	<0.001	n = 34 (100.0)	n = 33 (97.1)	0.003
General anesthesia	1 (1.0)	9 (20.0)		1 (2.9)	9 (27.3)	
Conscious sedation	96 (99.0)	36 (80.0)		33 (97.1)	24 (72.7)	

PCI during TAVI hospitalization	5 (5.3)	1 (6.5)	0.716	1 (3.1)	1 (3.0)	1.000
Extracorporeal membrane oxygenation (ECMO) TAVI support	0 (0.0)	1 (2.8)	0.273	0 (0.0)	1 (4.2)	0.421

SD – standard deviation, PCI – percutaneous coronary intervention, TAVI – transcatheter aortic valve implantation, THV – transcatheter heart valve, NA – not applicable.

Table S2. Binary logistic regression analysis of predictors for permanent pacemaker implantation

Predictor	Odds ratio	p-value
Valve type	0.329	0.247
Propensity score	6.328	0.408

Table S3. Post-procedural secondary outcomes

In-hospital outcomes n (%)	Unmatched cohort			Matched cohort		
	Myval (n = 97)	Portico (n = 47)	p-value	Myval (n = 34)	Portico (n = 34)	p-value
Access site complications	20 (21.1)	10 (22.7)	0.999	9 (27.3)	7 (21.2)	0.774
Local hematoma	12 (12.4)	4 (8.5)	0.683	4 (11.8)	2 (5.9)	0.673
Retroperitoneal hematoma	1 (1.0)	0 (0.0)	1.000	0 (0.0)	0 (0.0)	-
Dissection of the artery	2 (2.1)	1 (2.1)	1.000	0 (0.0)	1 (2.9)	1.000
Perforation of the artery	1 (1.0)	0 (0.0)	1.000	0 (0.0)	0 (0.0)	-
Pseudoaneurysm/AV-fistula	4 (4.1)	3 (6.4)	0.683	3 (8.8)	3 (8.8)	1.000
Arterial stenosis	3 (3.1)	2 (4.3)	0.661	2 (5.9)	1 (2.9)	1.000
Arterial stenosis severity						

Insignificant (< 50 %)	1 (33.3)	1 (50.0)	1.000	1 (50.0)	1 (100.0)	1.000
Significant, but no intervention was needed	1 (33.3)	1 (50.0)		1 (50.0)	0 (0.0)	
Significant, intervention needed	1 (33.3)	0 (0.0)		0 (0.0)	0 (0.0)	
Neurological complications	3 (3.2) (n = 94)	0 (0.0) (n = 44)	0.551	1 (3.0) (n = 33)	0 (0.0) (n = 33)	-
Transient ischemic attack	1 (1.0)	0 (0.0)	1.000	0 (0.0)	0 (0.0)	-
Ischemic cerebrovascular insult	2 (2.1)	0 (0.0)	1.000	1 (2.9)	0 (0.0)	-
Hemorrhagic cerebrovascular insult	0 (0.0)	0 (0.0)	-	0 (0.0)	0 (0.0)	-
Cardiac death etiology	2 (100.0)	0 (0.0)	1.000	0 (0.0)	0 (0.0)	-
Heart failure	1 (50.0)	0 (0.0)	-	0 (0.0)	0 (0.0)	-
Sudden cardiac death	1 (50.0)	0 (0.0)	-	0 (0.0)	0 (0.0)	-
Acute kidney injury	1 (1.0)	3 (6.4)	0.102	1 (2.9)	3 (8.8)	0.614
New LBBB	10 (10.3)	2 (4.3)	0.337	6 (17.7)	2 (5.9)	0.259
New atrial fibrillation	5 (5.2)	5 (10.6)	0.295	4 (11.8)	5 (14.7)	1.000
Duration of hospital stay after TAVI in days (median)	4	6		5	6	

AV – arterio-venous, LBBB – left-bundle branch block, MI – myocardial infarction, TAVI – transcatheter aortic valve implantation.

Table S4. Comparison of pre- and post-procedural hemodynamic parameters.

	**Unmatched cohort**			**Matched cohort**		
	**Pre-procedure**	**Post-procedure**	**p-value**	**Pre-procedure**	**Post-procedure**	**p-value**

**Myval THV series (Mean ± SD)**						
Aortic Vmax (m/s)	4.3 ± 0.5 (n = 85)	2.1 ± 0.5 (n = 93)	<0.001	4.2 ± 0.6 (n = 28)	1.9 ± 0.3 (n = 33)	<0.001
Aortic mean gradient (mm Hg)	45.1 ± 12.5 (n = 96)	11.1 ± 5.2 (n = 93)	<0.001	43.7 ± 13.1 (n = 34)	8.9 ± 2.5 (n = 33)	<0.001
AVA (cm^2)	0.7 ± 0.2 (n = 95)	1.8 ± 0.4 (n = 94)	<0.001	0.7 ± 0.3 (n = 32)	1.9 ± 0.4 (n = 34)	<0.001
LVEF (%)	58.2 ± 13.0 (n = 96)	59.9 ± 12.5 (n = 86)	0.030	51.7 ± 13.1 (n = 34)	54.1 ± 14.1 (n = 33)	0.087
**Portico THV (Mean ± SD)**						
Aortic Vmax (m/s)	3.9 ± 1.1 (n = 40)	2.0 ± 0.4 (n = 41)	<0.001	4.0 ± 1.0 (n = 33)	1.9 ± 0.4 (n = 32)	<0.001
Aortic mean gradient (mm Hg)	43.8 ± 10.7 (n = 41)	8.5 ± 4.5 (n = 38)	<0.001	44.0 ± 11.4 (n = 34)	8.1 ± 4.7 (n = 29)	<0.001
AVA (cm^2)	0.7 ± 0.2 (n = 42)	1.8 ± 0.5 (n = 44)	<0.001	0.7 ± 0.2 (n = 34)	1.8 ± 0.5 (n = 32)	<0.001
LVEF (%)	59.3 ± 10.7 (n = 39)	61.7 ± 11.2 (n = 40)	0.552	60.1 ± 10.0 (n = 34)	61.1 ± 11.4 (n = 31)	0.819

AVA – aortic valve area; LVEF – left ventricular ejection fraction; SD – standard deviation; THV – transcatheter heart valve, Vmax – maximal speed of blood in ascending aorta measured with continuous doppler ultrasound.

Table S5. Analysis of hemodynamic parameters by using repeated measure two-way ANOVA.

**Parameters**	**Between THV systems**		**Interaction Effect**		**Degrees of Freedom**	
	**p-value**	**F-statistics**	**p-value**	**F-statistics**	**Condition**	**Residual**

AVA (cm^2)	0.1743	1.89	0.1442	2.19	1	64
Aortic Mean Gradient (mm Hg)	0.6590	0.20	0.4680	0.53	1	66
Aortic Vmax (m/s)	0.6380	0.22	0.2900	1.14	1	66
LVEF (%)	0.0871	3.02	0.1783	1.85	1	66

AVA – aortic valve area; LVEF – left ventricular ejection fraction; THV – transcatheter heart valve, Vmax – maximal speed of blood in ascending aorta measured with continuous doppler ultrasound.

Table S6. Sex stratified analysis of outcomes post-TAVI.

In-hospital Outcomes, n (%)	Overall (n = 144)						Matched (n = 68)					
	Myval (n = 97)			Portico (n = 47)			Myval (n = 34)			Portico (n = 34)		
	Male (n = 52)	Female (n = 45)	p-value	Male (n = 16)	Female (n = 31)	p-value	Male (n = 21)	Female (n = 13)	p-value	Male (n = 15)	Female (n = 19)	p-value
Cardiac complications	n=50	n=45	0.0468	n=16	n=27	1.0000	n=20	n=13	0.3939	n=15	n=18	1.0000
1-Yes	0 (0)	4 (8.89)		1 (6.25)	3 (11.11)		0 (0)	1 (7.69)		1 (6.67)	2 (11.11)	
0-No	50 (100)	41 (91.11)		15 (93.75)	24 (88.89)		20 (100)	12 (92.31)		14 (93.33)	16 (88.89)	
Cardiac complications - new pericardial effusion	n=52	n=45	1.0000	n=16	n=31	1.0000	n=21	n=13	1.0000	n=15	n=19	1.0000
1-Yes	0 (0)	0 (0)		0 (0)	0 (0)		0 (0)	0 (0)		0 (0)	0 (0)	
0-No	52 (100)	45 (100)		16 (100)	31 (100)		21 (100)	13 (100)		15 (100)	19 (100)	
Cardiac complications – tamponade	n=52	n=45	1.0000	n=16	n=31	1.0000	n=21	n=13	1.0000	n=15	n=19	1.0000
1-Yes	0 (0)	0 (0)		0 (0)	1 (3.23)		0 (0)	0 (0)		0 (0)	1 (5.26)	
0-No	52 (100)	45 (100)		16 (100)	30 (96.77)		21 (100)	13 (100)		15 (100)	18 (94.74)	
Cardiac complications - annular rupture	n=52	n=45	0.4639	n=16	n=31	1.0000	n=21	n=13	1.0000	n=15	n=19	1.0000
1-Yes	0 (0)	1 (2.22)		0 (0)	0 (0)		0 (0)	0 (0)		0 (0)	0 (0)	
0-No	52 (100)	44 (97.78)		16 (100)	31 (100)		21 (100)	13 (100)		15 (100)	19 (100)	
Cardiac complications - valve embolization	n=52	n=45	1.0000	n=16	n=31	1.0000	n=21	n=13	1.0000	n=15	n=19	1.0000
1-Yes	0 (0)	0 (0)		0 (0)	1 (3.23)		0 (0)	0 (0)		0 (0)	0 (0)	
0-No	52 (100)	45 (100)		16 (100)	30 (96.77)		21 (100)	13 (100)		15 (100)	19 (100)	

Cardiac complications - improper valve position	n=52	n=45	1.0000	n=16	n=31	1.0000	n=21	n=13	1.00000	n=15	n=19	1.0000
1-Yes	0 (0)	0 (0)		1 (6.25)	1 (3.23)		0 (0)	0 (0)		1 (6.67)	1 (5.26)	
0-No	52 (100)	45 (100)		15 (93.75)	30 (96.77)		21 (100)	13 (100)		14 (93.33)	18 (94.74)	
Cardiac complications - conversion to heart surgery	n=52	n=45	1.0000	n=16	n=31	1.0000	n=21	n=13	1.0000	n=15	n=19	1.0000
1-Yes	0 (0)	0 (0)		0 (0)	1 (3.23)		0 (0)	0 (0)		0 (0)	0 (0)	
0-No	52 (100)	45 (100)		16 (100)	30 (96.77)		21 (100)	13 (100)		15 (100)	19 (100)	
Cardiac complications - Periprocedural MI (<72h)	n=52	n=45	0.0962	n=16	n=31	1.0000	n=21	n=13	0.3824	n=15	n=19	1.0000
1-Yes	0 (0)	3 (6.67)		0 (0)	0 (0)		0 (0)	1 (7.69)		0 (0)	0 (0)	
0-No	52 (100)	42 (93.33)		16 (100)	31 (100)		21 (100)	12 (92.31)		15 (100)	19 (100)	
Cardiac complications - spontaneous MI (>72h)	n=52	n=45	1.0000	n=16	n=31	1.0000	n=21	n=13	1.0000	n=15	n=19	1.0000
1-Yes	0 (0)	0 (0)		0 (0)	0 (0)		0 (0)	0 (0)		0 (0)	0 (0)	
0-No	52 (100)	45 (100)		16 (100)	31 (100)		21 (100)	13 (100)		15 (100)	19 (100)	
Access site complications	n=50	n=45	0.6050	n=16	n=28	1.0000	n=20	n=13	0.4251	n=15	n=18	1.0000
1-Yes	9 (18)	11 (24.44)		4 (25)	6 (21.43)		4 (20)	5 (38.46)		3 (20)	4 (22.22)	
0-No	41 (82)	34 (75.56)		12 (75)	22 (78.57)		16 (80)	8 (61.54)		12 (80)	14 (77.78)	
Access site complications - local hematoma	n=52	n=45	0.5640	n=16	n=31	0.5968	n=21	n=13	0.2740	n=15	n=19	1.0000
1-Yes	5 (9.62)	7 (15.56)		2 (12.5)	2 (6.45)		1 (4.76)	3 (23.08)		1 (6.67)	1 (5.26)	
0-No	47 (90.38)	38 (84.44)		14 (87.5)	29 (93.55)		20 (95.24)	10 (76.92)		14 (93.33)	18 (94.74)	
Access site complications - retroperitoneal hematoma	n=52	n=45	0.4639	n=16	n=31	1.0000	n=21	n=13	1.0000	n=15	n=19	1.0000
1-Yes	0 (0)	1 (2.22)		0 (0)	0 (0)		0 (0)	0 (0)		0 (0)	0 (0)	
0-No	52 (100)	44 (97.78)		16 (100)	31 (100)		21 (100)	13 (100)		15 (100)	19 (100)	
Access site complications - dissection of the artery	n=52	n=45	0.2126	n=16	n=31	1.0000	n=21	n=13	1.0000	n=15	n=19	1.0000
1-Yes	0 (0)	2 (4.44)		0 (0)	1 (3.23)		0 (0)	0 (0)		0 (0)	1 (5.26)	
0-No	52 (100)	43 (95.56)		16 (100)	30 (96.77)		21 (100)	13 (100)		15 (100)	18 (94.74)	
Access site complications - perforation of the artery	n=52	n=45	0.4639	n=16	n=31	1.0000	n=21	n=13	1.0000	n=15	n=19	1.0000

1-Yes	0 (0)	1 (2.22)		0 (0)	0 (0)		0 (0)	0 (0)		0 (0)	0 (0)	
0-No	52 (100)	44 (97.78)		16 (100)	31 (100)		21 (100)	13 (100)		15 (100)	19 (100)	
Access site complications - surgical wound infection	n=52	n=45	1.0000	n=16	n=31	1.0000	n=21	n=13	1.0000	n=15	n=19	1.0000
1-Yes	0 (0)	0 (0)		0 (0)	0 (0)		0 (0)	0 (0)		0 (0)	0 (0)	
0-No	52 (100)	45 (100)		16 (100)	31 (100)		21 (100)	13 (100)		15 (100)	19 (100)	
Access site complications - pseudoaneurysm/AV-fistula	n=52	n=45	0.6211	n=16	n=31	0.2640	n=21	n=13	1.0000	n=15	n=19	0.5714
1-Yes	3 (5.77)	1 (2.22)		2 (12.5)	1 (3.23)		2 (9.52)	1 (7.69)		2 (13.33)	1 (5.26)	
0-No	49 (94.23)	44 (97.78)		14 (87.5)	30 (96.77)		19 (90.48)	12 (92.31)		13 (86.67)	18 (94.74)	
Access site complications - arterial stenosis	n=52	n=45	0.5953	n=16	n=31	0.5412	n=21	n=13	1.0000	n=15	n=19	1.0000
1-Yes	1 (1.92)	2 (4.44)		0 (0)	2 (6.45)		1 (4.76)	1 (7.69)		0 (0)	1 (5.26)	
0-No	51 (98.08)	43 (95.56)		16 (100)	29 (93.55)		20 (95.24)	12 (92.31)		15 (100)	18 (94.74)	
Access site complications - lower limb ischemia	n=52	n=45	1.0000	n=16	n=31	1.0000	n=21	n=13	1.0000	n=15	n=19	1.0000
1-Yes	0 (0)	0 (0)		0 (0)	0 (0)		0 (0)	0 (0)		0 (0)	0 (0)	
0-No	52 (100)	45 (100)		16 (100)	31 (100)		21 (100)	13 (100)		15 (100)	19 (100)	
Access site complications - arterial stenosis severity												
1-insignificant (<50 %)	0	1		0	1		0	1		0	1	
2-significant, but not intervention needed	1	0		0	1		1	0		0	0	
3-significant, intervention needed	0	1		0	0		0	0		0	0	
Neurological complications	n=50	n=44	0.0988	n=16	n=28	1.0000	n=20	n=13	0.3939	n=15	n=18	1.0000
1-Yes	0 (0)	3 (6.82)		0 (0)	0 (0)		0 (0)	1 (7.69)		0 (0)	0 (0)	
0-No	50 (100)	41 (93.18)		16 (100)	28 (100)		20 (100)	12 (92.31)		15 (100)	18 (100)	
Neurological complications - TIA	n=52	n=45	0.4639	n=16	n=31	1.0000	n=21	n=13	1.0000	n=15	n=19	1.0000
1-Yes	0 (0)	1 (2.22)		0 (0)	0 (0)		0 (0)	0 (0)		0 (0)	0 (0)	
0-No	52 (100)	44 (97.78)		16 (100)	31 (100)		21 (100)	13 (100)		15 (100)	19 (100)	
Neurological complications - ischemic cerebrovascular insult	n=52	n=45	0.2126	n=16	n=31	1.0000	n=21	n=13	0.3824	n=15	n=19	1.0000
1-Yes	0 (0)	2 (4.44)		0 (0)	0 (0)		0 (0)	1 (7.69)		0 (0)	0 (0)	

0-No	52 (100)	43 (95.56)		16 (100)	31 (100)		21 (100)	12 (92.31)		15 (100)	19 (100)	
Hemorrhagic cerebrovascular insult	n=52	n=45	1.0000	n=16	n=31	1.0000	n=21	n=13	1.0000	n=15	n=19	1.0000
1-Yes	0 (0)	0 (0)		0 (0)	0 (0)		0 (0)	0 (0)		0 (0)	0 (0)	
0-No	52 (100)	45 (100)		16 (100)	31 (100)		21 (100)	13 (100)		15 (100)	19 (100)	
Bleeding	n=50	n=45	0.7043	n=16	n=27	1.0000	n=20	n=13	0.5473	n=15	n=17	1.0000
1-Yes	3 (6)	4 (8.89)		1 (6.25)	2 (7.41)		1 (5)	2 (15.38)		1 (6.67)	2 (11.76)	
0-No	47 (94)	41 (91.11)		15 (93.75)	25 (92.59)		19 (95)	11 (84.62)		14 (93.33)	15 (88.24)	
Bleeding - minor	n=52	n=45	0.6605	n=16	n=31	0.3404	n=21	n=13	0.1390	n=15	n=19	0.4412
1-Yes	2 (3.85)	3 (6.67)		1 (6.25)	0 (0)		0 (0)	2 (15.38)		1 (6.67)	0 (0)	
0-No	50 (96.15)	42 (93.33)		15 (93.75)	31 (100)		21 (100)	11 (84.62)		14 (93.33)	19 (100)	
Bleeding - major	n=52	n=45	1.0000	n=16	n=31	0.5412	n=21	n=13	1.0000	n=15	n=19	0.4920
1-Yes	1 (1.92)	0 (0)		0 (0)	2 (6.45)		1 (4.76)	0 (0)		0 (0)	2 (10.53)	
0-No	51 (98.08)	45 (100)		16 (100)	29 (93.55)		20 (95.24)	13 (100)		15 (100)	17 (89.47)	
Bleeding - life-threatening	n=52	n=45	0.4639	n=16	n=31	1.0000	n=21	n=13	1.0000	n=15	n=19	1.0000
1-Yes	0 (0)	1 (2.22)		0 (0)	0 (0)		0 (0)	0 (0)		0 (0)	0 (0)	
0-No	52 (100)	44 (97.78)		16 (100)	31 (100)		21 (100)	13 (100)		15 (100)	19 (100)	
Pacemaker implantation after TAVI	n=50	n=45	1.0000	n=16	n=27	0.3437	n=20	n=13	0.5473	n=15	n=17	0.6454
1-Yes	5 (10)	5 (11.11)		3 (18.75)	2 (7.41)		1 (5)	2 (15.38)		3 (20)	2 (11.76)	
0-No	45 (90)	40 (88.89)		13 (81.25)	25 (92.59)		19 (95)	11 (84.62)		12 (80)	15 (88.24)	
Death during TAVI procedure	n=50	n=45	1.0000	n=16	n=26	1.0000	n=20	n=13	1.0000	n=15	n=17	1.0000
1-Yes	0 (0)	0 (0)		0 (0)	0 (0)		0 (0)	0 (0)		0 (0)	0 (0)	
0-No	50 (100)	45 (100)		16 (100)	26 (100)		20 (100)	13 (100)		15 (100)	17 (100)	
Cause of death												
1=cardiac	0 (0)	0 (0)		0 (0)	0 (0)		0 (0)	0 (0)		0 (0)	0 (0)	
Cardiac death etiology												
1=MI	0 (0)	0 (0)		0 (0)	0 (0)		0 (0)	0 (0)		0 (0)	0 (0)	
3=heart failure	0 (0)	0 (0)		0 (0)	0 (0)		0 (0)	0 (0)		0 (0)	0 (0)	
7=sudden cardiac death	0 (0)	0 (0)		0 (0)	0 (0)		0 (0)	0 (0)		0 (0)	0 (0)	
Death after TAVI until hospital discharge	n=49	n=44	0.2211	n=16	n=25	1.0000	n=20	n=13	1.0000	n=15	n=15	1.0000

1-Yes	0 (0)	2 (4.55)		0 (0)	0 (0)		0 (0)	0 (0)		0 (0)	0 (0)	
0-No	49 (100)	42 (95.45)		16 (100)	25 (100)		20 (100)	13 (100)		15 (100)	15 (100)	
Cause of death	n=0	n=2		n=0	n=0		n=0	n=0		n=0	n=0	
1=cardiac	0 (0)	2 (100)		0 (0)	0 (0)		0 (0)	0 (0)		0 (0)	0 (0)	
2=non-cardiac	0 (0)	0 (0)		0 (0)	0 (0)		0 (0)	0 (0)		0 (0)	0 (0)	
Cardiac death etiology	n=0	n=2		0 (0)	0 (0)		0 (0)	0 (0)		0 (0)	0 (0)	
1=MI	0 (0)	0 (0)		0 (0)	0 (0)		0 (0)	0 (0)		0 (0)	0 (0)	
2=tamponade	0 (0)	0 (0)		0 (0)	0 (0)		0 (0)	0 (0)		0 (0)	0 (0)	
3=heart failure	0 (0)	1 (50)		0 (0)	0 (0)		0 (0)	0 (0)		0 (0)	0 (0)	
4=pulmonary embolism	0 (0)	0 (0)		0 (0)	0 (0)		0 (0)	0 (0)		0 (0)	0 (0)	
5=aortic dissection	0 (0)	0 (0)		0 (0)	0 (0)		0 (0)	0 (0)		0 (0)	0 (0)	
6=prosthesis related	0 (0)	0 (0)		0 (0)	0 (0)		0 (0)	0 (0)		0 (0)	0 (0)	
7=sudden cardiac death	0 (0)	1 (50)		0 (0)	0 (0)		0 (0)	0 (0)		0 (0)	0 (0)	
8=consequences of rescue procedures	0 (0)	0 (0)		0 (0)	0 (0)		0 (0)	0 (0)		0 (0)	0 (0)	
9=vascular complication	0 (0)	0 (0)		0 (0)	0 (0)		0 (0)	0 (0)		0 (0)	0 (0)	
Other complications – acute kidney injury	n=52	n=45	0.4639	n=16	n=31	1.0000	n=21	n=13	0.3824	n=15	n=19	1.0000
1-Yes	0 (0)	1 (2.22)		1 (6.25)	2 (6.45)		0 (0)	1 (7.69)		1 (6.67)	2 (10.53)	
0-No	52 (100)	44 (97.78)		15 (93.75)	29 (93.55)		21 (100)	12 (92.31)		14 (93.33)	17 (89.47)	
Other complications – new LBBB	n=52	n=45	0.0052	n=16	n=31	1.0000	n=21	n=13	0.0214	n=15	n=19	1.0000
1-Yes	1 (1.92)	9 (20)		1 (6.25)	1 (3.23)		1 (4.76)	5 (38.46)		1 (6.67)	1 (5.26)	
0-No	51 (98.08)	36 (80)		15 (93.75)	30 (96.77)		20 (95.24)	8 (61.54)		14 (93.33)	18 (94.74)	
Other complications – new atrial fibrillation	n=52	n=45	0.3685	n=16	n=31	0.3202	n=21	n=13	1.0000	n=15	n=19	0.6343
1-Yes	4 (7.69)	1 (2.22)		3 (18.75)	2 (6.45)		3 (14.29)	1 (7.69)		3 (20)	2 (10.53)	
0-No	48 (92.31)	44 (97.78)		13 (81.25)	29 (93.55)		18 (85.71)	12 (92.31)		12 (80)	17 (89.47)	
Days of hospital stay after TAVI, Days, (Median)	4.00	5.00		6	6		4	5		6	6	
30-day mortality	n=51	n=45	0.2171	n=16	n=31	1.0000	n=20	n=13	1.0000	n=15	n=19	1.0000
1-Yes	0 (0)	2 (4.44)		0 (0)	1 (3.33)		0 (0)	0 (0)		0 (0)	1 (5.26)	

0-No	51 (100)	43 (95.56)		16 (100)	29 (96.67)		20 (100)	13 (100)		15 (100)	18 (94.74)	
1-year mortality	n=51	n=45	1.0000	n=16	n=30	0.5415	n=20	n=13	1.0000	n=15	n=19	0.4920
1-Yes	3 (5.88)	2 (4.44)		0 (0)	3 (10)		0 (0)	0 (0)		0 (0)	2 (10.53)	
0-No	48 (94.12)	43 (95.56)		16 (100)	27 (90)		20 (100)	13 (100)		15 (100)	17 (89.47)	

AV – arterio-venous; LBBB – left bundle branch block; MI – myocardial infarction; TAVI – transcatheter aortic valve implantation; TIA – transient ischemic attack.
